# Oral microbiome patterns of dental caries in Kazakhstani adolescents

**DOI:** 10.1590/1678-7757-2025-0476

**Published:** 2025-11-17

**Authors:** Argul ISSILBAYEVA, Zharkyn JARMUKHANOV, Samat KOZHAKHMETOV, Yermekbayeva BAKYTGUL, Laura CHULENBAYEVA, Graciela MUNIZ-TERRERA, Masae FURUKAWA, Hiroki NIKAWA, Adil SUPIYEV, Almagul KUSHUGULOVA, Anara ZHUMADILOVA

**Affiliations:** 1 Nazarbayev University National Laboratory Astana Center for Life Sciences Astana Kazakhstan Nazarbayev University, National Laboratory Astana, Center for Life Sciences, Astana, Kazakhstan.; 2 Ohio University Heritage College of Osteopathic Medicine Athens Ohio USA Ohio University, Heritage College of Osteopathic Medicine, Athens, Ohio, USA.; 3 National Institutes of Health Eunice Kennedy Shriver National Institute of Child Health and Human Development Bethesda MD USA National Institutes of Health (NIH), Eunice Kennedy Shriver National Institute of Child Health and Human Development (NICHD), Bethesda, MD, USA.; 4 Hiroshima University Graduate School of Biomedical and Health Sciences Hiroshima Japan Hiroshima University, Graduate School of Biomedical and Health Sciences, Hiroshima, Japan.

**Keywords:** Oral microbiome, DMFT in Kazakhstan, Adolescents’ oral health, Microbiome patterns

## Abstract

**Objective:**

The oral microbiome is one of the most complex microbial ecosystems in the host. This study aimed to investigate and characterize the oral microbiome composition in Kazakhstani adolescents associated with dental caries.

**Methodology:**

The study included 312 adolescents, with 241 individuals presenting with caries and 71 caries-free, aged 12-15 years. Dental caries assessment was performed using DMFT (Decayed, missed, filled teeth) index. Oral samples were collected, and 16S rRNA (16S ribosomal ribonucleic acid) gene sequencing targeting the V3-V4 hypervariable regions on an Illumina MiSeq platform was performed to profile the microbial communities. Functional metagenomic predictions were generated using PICRUSt2 v2.5.0, using the KEGG database for bacterial pathway abundance estimation. Data analysis was conducted using Python 3.9.16 and R 4.2.2.

**Results:**

The alpha diversity was insignificant, while beta diversity analysis demonstrated clear distinctions by Bray-Curtis (F=2.5, p=0.003) and weighted UniFrac distances (F=4.4, p=0.002). The *Neisseria* and *Prevotella* genera, and *Gammaproteobacteria* class showed significant associations with dental caries (MaAsLin2 p≤0.05, LDA≥2), stronger predictive power (AUC=0.65, F1=0.83), and higher predicted functional activity through glutathione metabolism, RNA degradation, and unsaturated fatty acid metabolism pathways.

**Conclusions:**

This study identified specific oral microbiome patterns associated with dental caries in Kazakhstani adolescents, revealing interactions between key bacterial taxa and metabolic pathways.

## Graphical abstract

**Figure:**
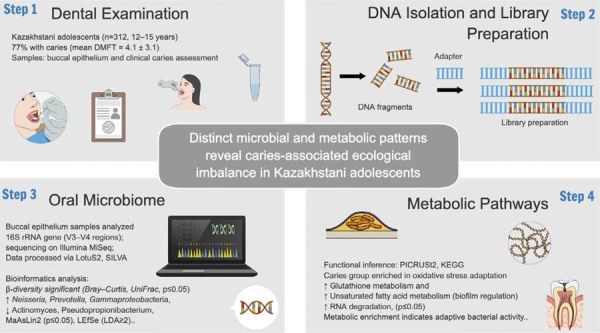

## Introduction

The bacterial community inhabiting the oral cavity is exceptionally diverse and one of the most complex microbial ecosystems in the human body, playing a crucial role in oral health and the development of dental caries.^1-3^ Hence, adolescence represents a critical period for oral health,^4,5^ characterized by significant changes in dietary habits, lifestyle, and hormonal balance that may influence oral microbiome composition.^6^ Among oral health issues, caries is a significant concern,^2^ particularly affecting individuals since childhood.^2,3^ Despite the high prevalence of dental caries in various age groups, adolescence demonstrates the highest rates of permanent tooth caries, establishing a persistent prevalence level according to WHO,^4^ which extends beyond dental health and potentially impacts the broader development of individuals.

While past research concentrated on specific bacteria, contemporary advanced sequencing methods, mainly 16S rRNA sequencing, have unraveled an oral ecosystem containing hundreds of bacterial species, shifting our comprehension of caries-associated bacterial communities and their influence on oral cavity health.^2^ Diverse bacterial populations create intricate layers known as biofilms, which generally exist in harmony with the host.^3^ This balance can be destabilized by several factors, such as diet, oral hygiene habits, environmental conditions, host-health-related factors (including reduced salivary secretion, compromised immune response, and altered buffering capacity), lifestyle, and socio-economic factors.^2-4^ All these factors create an environment leading to a pH decrease, where acid-producing and acid-resistant microorganisms thrive, fundamentally altering the natural balance between mineral loss and replacement in dental tissues and transforming a healthy oral microbiome into one that promotes caries.^3^

Although numerous studies have investigated oral microbiome composition in early childhood,^7,8^ comprehensive studies of oral microbial communities during adolescence remain limited.^1^ Studies of adolescent caries have been conducted in East Asia,^7,9^ the Middle East,^10^ Europe,^11,12^ and the USA.^13^ Yet, there is a notable absence of studies from Central Asian regions, creating a significant geographical gap in caries data.

Thus, understanding these microbial dynamics during adolescence becomes particularly crucial as teenage years often establish long-term oral health patterns.^5^

The objective of this study is to investigate and characterize the oral microbiome patterns and microbial diversity in Kazakhstani adolescents associated with dental caries, aiming to provide essential insights into microbial balance and its relationship with caries risk in this population. Additionally, the study aims to explore the potential role of metabolic pathways associated with key bacterial taxa in influencing caries progression.

## Methodology

### Ethical aspects

The research protocol received approval from Nazarbayev University’s Centre for Life Sciences Research Ethics Committee (protocol No. 20, September 22, 2017,^14^ and protocol No. 03-2024, October 2, 2024). Parents received comprehensive study information in both Kazakh and Russian. For participants under 16 years of age, written parental consent and participant assent were obtained in accordance with ethical guidelines.

### Recruitment of study participants

This study is a subset of the Kazakhstan Adolescent Health Study (KAHS), which was conducted by Nazarbayev University’s Laboratory of Epidemiology in collaboration with University College London and adapted WHO’s Health Behavior in School-age children study protocols and Child Dental Health Survey methodology. It included 2,149 adolescents (2014-2015).^14^ Of these, 1,256 participants refused sample collection for sequencing, and 414 did not meet our inclusion criteria, resulting in 479 participants who met inclusion/exclusion criteria and provided oral samples. After DNA extraction and quality and quantity control, 312 samples met the requirements for sequencing conditions (Supplementary Figure 1).

**Figure 1:**
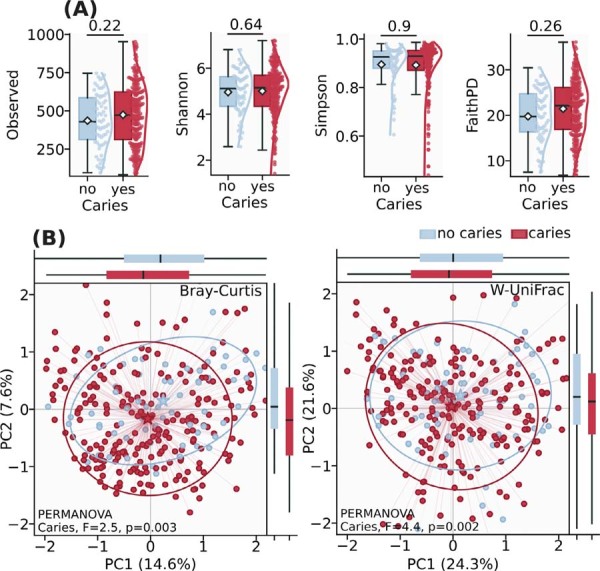
Comparative analysis of biodiversity of oral microbiome composition in no caries and caries groups in adolescents. (A) Within-sample (alpha) diversity assessed using the Observed, Shannon, Simpson, and Faith indices at the ASV level. Generalized linear model (GLM). (B) Principal coordinate (PCoA) analysis ordination depicting between-sample (beta) diversity measured using Bray-Curtis and Weighted (W)-Uni-Frac distances on Hellinger-transformed data at the ASV level. PERMANOVA grouping test with 999 permutations.

This approach balanced the resource-intensive nature of 16S rRNA sequencing with the need for sufficient statistical power. Random sampling ensured the representativeness of the selected cohort and allowed for an unbiased investigation of the relationship between oral microbiome composition and caries status. This cross-sectional design offered a snapshot of microbiome patterns in the chosen age group, enabling the exploration of links between caries and microbiome composition.

Participants were adolescents aged 12-15 years, both male and female, with normal growth and development, who had resided in Kazakhstan for at least 10 years. Exclusion criteria included a history of professional dental cleaning within the past month; the presence of periodontal disease or orthodontic appliances; special dietary restrictions such as physician-prescribed diets related to gastrointestinal diseases or other health conditions that could affect the oral or gut microbiome; use of antibiotics, probiotics, or antiviral medications in the past 3 months; and a history of cancer, autoimmune disorders, severe somatic conditions, parasitic infections, or any regular medication or biologically active supplement use within the last 3 months.

The data collection encompassed sociodemographic factors (age, gender, ethnicity, parental status, education, material deprivation), dietary habits (sugar consumption), and oral health practices (tooth brushing frequency, dental visit patterns). Structured questionnaires, anthropometric measurements, and clinical exams were conducted by trained healthcare professionals.^14^

Clinical assessments included dental examinations following WHO guidelines^15^ using natural daylight from windows, supplemented by standard room lighting and examiner-worn headlamps, with patients seated in conventional chairs. The examination tools included single-use sterilized instruments: an illuminated oral mirror and a standardized periodontal probe (0.5 mm ball-tip diameter). The findings were documented on individual assessment forms. For dental caries assessment, the DMFT index, indicating the number of decayed, missing, and filled teeth (range 0-28, excluding third molars, with 0 indicating no dental caries experience and 28 indicating all permanent teeth are decayed, missing, or filled), with D3 threshold criteria,^16^ was used. A higher DMFT score generally indicates poorer oral health. The dental health assessment methodology was based on modified Child Dental Health Survey (CDHS) protocols, featuring specially adapted survey instruments that collected data from both participating students and their parents.^17^ The findings were documented on individual assessment forms.

### Microbiome investigation

#### Sample collection

Participants followed specific pre-sampling protocols, including a 12-hour suspension of oral hygiene practices and a 2-hour abstention from food and beverage consumption before sample collection. The sampling methodology adhered to the standardized Human Microbiome Project Core Protocol.^18^ Multiple oral sites were sampled, with saliva collected from the oral floor using calibrated pipettes. Soft tissue samples were obtained from six distinct sites (tongue dorsum, hard palate, buccal surfaces, attached gingiva, palatine tonsils, and throat) using specialized ZymoBIOMICS™ DNA/RNA Shield Swab Collection systems. Both supragingival and subgingival plaque samples were collected from at least four molar teeth using Gracey curettes.

#### DNA isolation

Genomic material was isolated using the ZymoBIOMICS™ DNA Miniprep Kit. Quality assessment of extracted DNA was performed through 1% agarose gel electrophoresis, with concentration measurements conducted using an Invitrogen Qubit 3.0 Fluorometer, and sterile water serving as a negative control.

#### Microbiome sequencing

The 16S rRNA gene sequencing was conducted at the Laboratory of Microbiome, National Laboratory Astana, Nazarbayev University, utilizing the Illumina MiSeq platform and targeting the V3-V4 hypervariable regions. Sequence processing employed the LotuS2 pipeline, including demultiplexing, quality filtering, and dereplication. Chimeric sequences were removed using UCHIME algorithms, and taxonomic classification was performed using the SILVA database with UPARSE clustering. Total reads processed: 65,503,292. After filtering, 5,111 ASV’s (23,131,381 reads) remained in the matrix.

## Statistical analysis and data processing

Data analysis was performed using Python 3.9.16 and R 4.2.2. Statistical comparisons utilized Mann-Whitney U tests, Kruskal-Wallis H tests, Chi2 tests, Spearman correlation, or general multivariate regression models where appropriate, using SciPy 1.13.1 and Stats models 0.14.12. All analyses were conducted while adjusting for child age, gender, and region.

To identify microbial community patterns and diversity, multiple approaches were employed. Alpha diversity was assessed using Shannon, Simpson, Faith, and Observed indices, while beta diversity was evaluated using Bray-Curtis and weighted UniFrac distances with the PERMANOVA test (9,999 permutations) using scikit-bio 0.6.0. Community composition was visualized through PCoA on Hellinger-transformed data, and RDA analysis was performed using vegan 2.6.4 with scaling 2.

Taxonomic tables were generated using the LotuS2 pipeline. Data were not rarefied, and relative abundance was determined using the total-sum-scaling (TSS) method.

To identify differentially abundant taxa, a combined approach using MaAsLin2 (p≤0.05) and LEfSe (LDA>2) was implemented. Only features with at least 25% prevalence were considered for comparison, and multiple comparison corrections employed the FDR-BH methodology, except when comparing significantly differentially abundant parameters and descriptive statistics.

Feature importance was evaluated using gradient boosting decision trees and logistic or linear l2-regularized regression on leave-one-out cross-validation (scikit-learn 1.2.2, LightGBM 3.3.5). All visualizations were created using Matplotlib and Seaborn libraries.

Functional metagenomic predictions were generated using PICRUSt2 v2.5.0, employing a reference tree with an NSTI cutoff of 2. Metabolic pathway analysis utilized the KEGG database for bacterial pathway abundance estimation. Functional pathways were considered differentially abundant when 95% confidence intervals (CIs) for the difference between means did not overlap and p≤0.05. Identical processing methods were applied to all samples.

## Results

The overall sequenced sample of 312 adolescents included 46.2 % male and 53.8% female participants, with an average age of 13.3±1.4 years (see Table 1 for descriptive characteristics of the analytical sample). Caries accounted for 77.2%, and only 22.8% of the recruited adolescents did not have caries; the total DMFT index was 4.1±3.1.

**Table 1:** Analysis of the clinical-demographic data.

Parameters	All	No Caries	Caries	p-value
		(DMF=0)	(DMF>0)	
		(n=71)	(n=241)	
Age (years)				
Mn±Sd	13.3±1.4	12.8±1.0	13.5±1.4	0.002^a^*
DMFT (index)				
Mn±S	-	-	4.1±3.1	-
DMF, n (%)				
Decayed			139 (57.7%)	-
Missed			21 (8.7%)	-
Filled			81 (33.6%)	-
Sex, n (%)				
Male	144 (46.2%)	38 (53.5%)	106 (44.0%)	0.18^b^
Female	168 (53.8%)	33 (46.5%)	135 (56.0%)	
Ethnicity, n (%)				
Kaz	216 (69.2%)	37 (52.1%)	179 (74.3%)	<0.001^c^*
Rus	61 (19.6%)	17 (23.9%)	44 (18.3%)	
Other	35 (11.2%)	17 (23.9%)	18 (7.5%)	
Region, n (%)				
Astana	89 (28.5%)	17 (23.9%)	72 (29.9%)	<0.001^c^*
Kokshetau	88 (28.2%)	36 (50.7%)	52 (21.6%)	
Semey	57 (18.3%)	4 (5.6%)	53 (22.0%)	
Oskemen	78 (25.0%)	14 (19.7%)	64 (26.6%)	

^a^Mann-Whitney U rank test

^b^Fisher exact test

^c^Chi-square test

The comparative analysis of clinical and demographic data in the two study groups demonstrated unequal representation by age, ethnicity, and region (Table 1). The age of participants with caries (13.5±1.4 years) was significantly higher than that of those without caries (12.8±1.0 years, p=0.002). Regarding gender distribution, the caries group (DMF>0) had a higher proportion of females (56.0%) compared to the no caries group (DMF=0) (46.5%), though this difference was not statistically significant (p=0.18). The data also showed significant differences in the distribution of participants by ethnicity and region. Kazakh participants made up a higher proportion of the caries group (73.9%) compared to the no caries group (49.3%, p<0.001). Similarly, participants from the Astana region had a higher caries prevalence (29.9%) than those from other areas.

Analysis of alpha diversity did not demonstrate any significant differences in species richness or evenness, p=0.074 (Shannon), dominance-based diversity, p=0.99 (Simpson), species count, p=0.32 (Observed), or phylogenetic diversity, p=0.26 (Faith), (Figure 1A). In contrast, beta diversity analysis revealed significant differences between microbial communities, precisely compositional dissimilarities by Bray-Curtis (PERMANOVA, F=2.5, p=0.003), with PcoA accounting for 22.2% of total variation (PC1=14.6%, PC2=7.6%), and phylogenetic structure of the microbial communities by weighted UniFrac distances (PERMANOVA: F=4.4, p=0.002), with PcoA explaining 45.9% of total variation (PC1=24.3%, PC2=21.6%). The 95% confidence ellipses showed partial overlap between groups, indicating shared bacterial community features despite overall compositional differences (Figure 1B).

**Figure 2:**
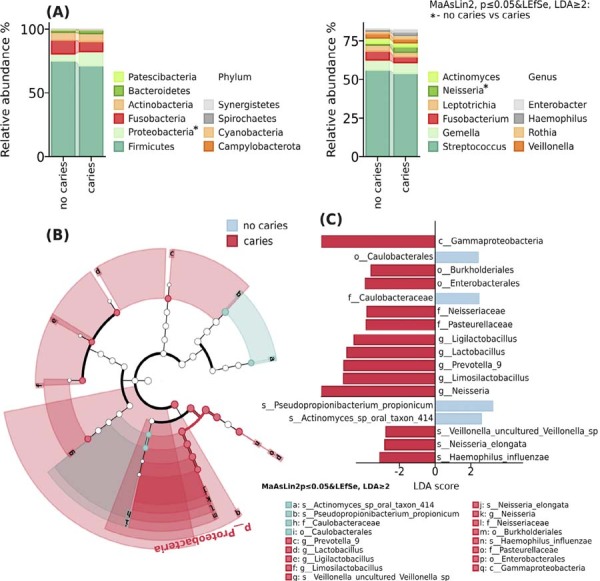
markers of oral microbiome composition in no caries and caries groups in adolescents. (A) Barplot of the mean of the top 10 most abundant taxa at the phylum and genus level by group. Significantly differentially abundant taxa are marked with (*). (B) Cladogram of taxonomic markers plotted using LEfSe tools. (C) Effect size barplot of taxonomic markers. LEfSe LDA. (A-C) MaAsLin, p ≤ 0.05 & LEfSe, LDA score ≥ 2. LefSe analysis identified significant taxonomic differences in the oral microbiome between caries and caries-free groups (MaAsLin2 p≤0.05, LDA≥2). The caries group showed significant enrichment of multiple taxa (LDA score≥2), including class Gammaproteobacteria, orders Burkholderiales and Enterobacterales, families *Neisseriaceae* and *Pasteurellaceae*, genera *Ligilactobacillus*, *Lactobacillus*, *Prevotella_9*, *Limosilactobacillus*, and *Neisseria*, as well as species *Veillonella* uncultured *Veillonella* sp, *Neisseria elongata*, and *Haemophilus influenzae*. In contrast, the caries-free group was characterized by a higher abundance of order *Caulobacterales*, family *Caulobacteriaceae*, and species *Pseudopropionibacterium propionicum* and *Actinomyces* sp. oral taxon 414, suggesting distinct microbial signatures associated with caries status, (Figure 2B&C).

Next, the relative abundance analysis revealed distinct microbial compositions between caries and non-caries adolescent groups (MaAsLin2, p≤0.05, LEfSe, LDA≥2), (Figure 2 and Supplementary Figure 2B). Among the top ten most abundant taxa, Firmicutes dominated at the phylum level (~60-70%), followed by Proteobacteria (which showed significant differences between groups, p=0.013, LDA=3.47), Fusobacteria, Actinobacteria, and Bacteroidetes. Less abundant phyla included Synergistetes, Spirochaetes, Cyanobacteria, and Campylobacterota (<5% relative abundance) (Figure 2А). At the class level, Bacilli were predominant, while Gammaproteobacteria demonstrated significant variation between groups, p=0.003, LDA=3.5. Order level analysis identified significant differences in Burkholderiales, p=0.01, LDA=3.32 and Enterobacterales, p=0.03, LDA=3.64 taxa. Family-level assessment revealed significant variations in *Neisseriaceae*, p=0.01, LDA=3.35 and Pasteurellaceae, p=0.03, LDA=3.41 abundances between groups. At the genus level, Streptococcus was the most abundant (>50% relative abundance) in both groups, with *Neisseria* (p=0.005, LDA=3.33), showing significant differences between caries and non-caries groups. Species-level analysis demonstrated that various *Streptococcus* species (S. mitis, S. infantis, S. sanguinis) were dominant members of the oral microbiota, with distinct distribution patterns between the study groups, p-values are available in Supplementary Table 1, Supplementary Figure 2A.

LefSe analysis identified significant taxonomic differences in the oral microbiome between caries and caries-free groups (MaAsLin2 p≤0.05, LDA≥2). The caries group showed significant enrichment of multiple taxa (LDA≥2), including class Gammaproteobacteria; orders Burkholderiales and Enterobacterales; families *Neisseriaceae* and *Pasteurellaceae*; genera *Ligilactobacillus*, *Lactobacillus*, *Prevotella_9*, *Limosilactobacillus*, and *Neisseria*; as well as uncultured *Veillonella* sp., *Neisseria elongata*, and *Haemophilus influenzae*. In contrast, the caries-free group was characterized by higher abundance of order Caulobacterales, family Caulobacteriaceae, and species *Pseudopropionibacterium propionicum* and *Actinomyces* sp. oral taxon 414, suggesting distinct microbial signatures associated with caries status (Figure 2B and C).

**Figure 3:**
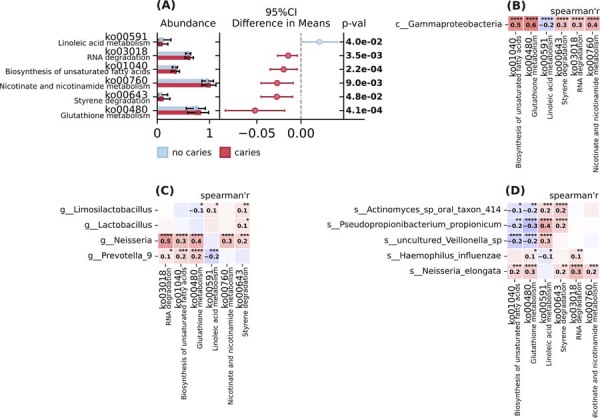
Functional markers of oral microbiome composition in adolescents' no caries and caries groups. (A) Confidence plot of functional markers. 95% confidence interval (CI) for the difference between group means, p≤ 0.05 & non-overlapping CI. (B-D): Correlation between significant taxonomic and functional markers. Spearman's rho, p≤0.05. (B) at class level; (C) at genus level; (D) at species level. *p≤ 0.05, **p≤ 0.01, ***p≤ 0.001, ****p≤ 0.0001.

Metabolic pathway analysis revealed significant differences between caries and no caries groups (Figure 3B). The glutathione metabolism pathway (p=4.1e-4), styrene degradation (p=4.8e-02), nicotinate and nicotinamide metabolism (p=9.0e-03), RNA degradation (p=3.5e-3), and biosynthesis of unsaturated fatty acids (p=2.2e-4) pathways were significantly enriched in the caries group, while linoleic acid metabolism was enriched in the no caries group (p=4.0e-02), (Figure 3A). Correlation analysis demonstrated that *g_Neisseria*, s*_Neisseria_elongata and c_Gammaproteobacteria* exhibited significant positive correlations with multiple pathways, particularly RNA degradation, biosynthesis of unsaturated fatty acids, glutathione metabolism, styrene degradation, and nicotinate and nicotinamide metabolism. Species *Actinomyces* sp. oral taxon 414 and *Pseudopropionibacterium propionicum* showed negative correlations with biosynthesis of unsaturated fatty acids and glutathione metabolism pathways and positive correlations with styrene degradation and linoleic acid metabolism. g_*Prevotella_9* displayed significant positive correlations with glutathione metabolism and biosynthesis of unsaturated fatty acids (r=0.2, p<0.001) and RNA degradation (r=0.1, p<0.05), and a negative correlation with linoleic acid metabolism (Figure 3B, C, D).

**Figure 4:**
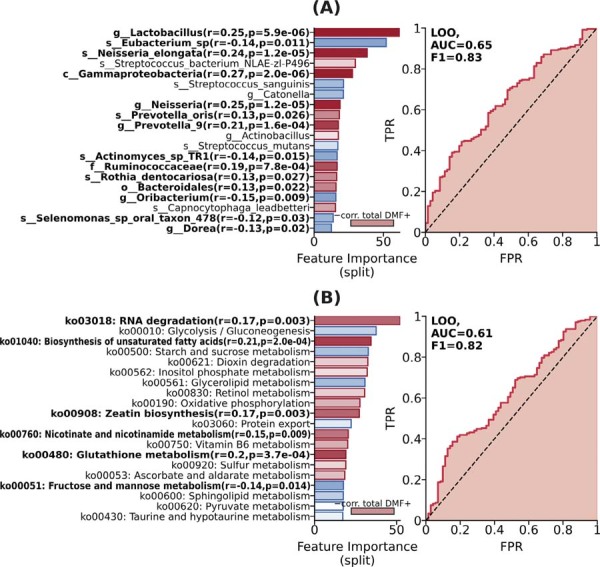
Analysis of the predictive power and importance of taxonomic and functional features of the oral microbiome in discriminating between no caries and caries groups in adolescents. (A-B) Barplot of top 20 predictive features and prognostic significance of no caries vs. caries classification models: (A) using taxonomic data; (B) using functional data. Features significantly correlated with the total number of affected by caries teeth (total DMF) are highlighted in bold. Gradient Boosting (GBDT) on leave-one-out cross-validation, no parameter tuning. TPR=True Positive Rate, FPR=False Positive Rate, AUC=Area Under Curve, F1=F1 Score. AUC summarizes the area under the TPR-FPR curve and relates to the probability that the prognostic model will correctly discriminate between classes. AUC>0.5 indicates non-random classification. F1 score indicates the harmonic mean of precision and recall. F1 ranges from 0 to 1, where 1 is a perfect classification.

Prognostic analysis showed moderately significant predictive performance on cross-validation. Figure 4 shows the most important features for classification, with characteristics that also correlate significantly with total DMFT highlighted in bold. Among bacterial taxa, g_*Lactobacillus*, s_*Neisseria_elongata*, and c_Gammaproteobacteria showed the strongest predictive power and significant correlations with caries. Other taxa correlated with caries in the model included g_*Neisseria*, g_*Prevotella_9*, s_*Prevotella oris*, f_ *Ruminococcaceae*, s_*Rothia dentocariosa*, and o_*Bacteroidales*. In contrast, taxa associated with the no-caries state included s_*Eubacterium* sp., s_ *Actinomyces* sp. TR1, g_ *Oribacterium*, and g_*Dorea*, with s_*Eubacterium* sp. showing the strongest predictive power. Overall, the model demonstrated good predictive performance for bacterial taxa (AUC=0.65, F1=0.83), suggesting their potential as bacterial markers for caries (Figure 4A).

Analysis of metabolic pathways revealed significant associations of several KEGG pathways between caries and no-caries groups, with RNA degradation (r=0.17, p=0.003), biosynthesis of unsaturated fatty acids (r=0.21, p=2.0e-04), zeatin biosynthesis (r=0.17, p=0.003), nicotinate and nicotinamide metabolism (r=0.15, p=0.009), and glutathione metabolism (r=0.2, p=3.7e-04) showing significant positive correlations with caries. RNA degradation and biosynthesis of unsaturated fatty acids showed the strongest predictive power towards caries. In contrast, in the model, fructose and mannose metabolism (r=-0.14, p=0.014) demonstrated a significant negative correlation with caries. The pathway-based prediction model achieved moderate performance (AUC=0.61, F1=0.82), indicating that functional metabolic changes are associated with caries (Figure 4B). Overall, feature importance scores and correlation analyses suggest that specific bacterial taxa show stronger predictive value than metabolic pathways.

Our RDA analysis demonstrated a significant influence of geographical region on oral microbiome composition (R^2^adj=0.0586, p=0.001). However, we acknowledge that our sample size for regional comparisons was limited, and these results should be interpreted with caution. Detailed regional comparisons of bacterial taxa abundances and their associations with caries status are provided in the Supplementary Materials (Supplementary Table 2, Supplementary Figure 2B), suggesting potential regional variations in oral microbiome patterns. Further studies with larger sample sizes and more diverse cohorts from each region would be beneficial.

## Discussion

Dental caries remains one of the most prevalent chronic diseases globally, affecting approximately 2.3 billion people worldwide.^19^ According to the WHO, the burden is particularly significant among children and adolescents with untreated caries in primary and permanent teeth.^4^ The development of dental caries involves a complex process resulting from disrupted microbial balance (dysbiosis) rather than from individual pathogenic species.^20^ Research demonstrates that acid-producing oral bacteria play a critical role in caries development through enamel demineralization, suggesting potential preventive strategies that target bacterial acid production.^21^ However, recent research indicates that dental caries is not limited to traditionally studied bacteria such as *Streptococcus mutans* and Lactobacillus; understanding this complex disease requires investigating the wider microbial community and its interactions in the caries process. Despite the high prevalence of dental caries across different age groups, comprehensive microbiome studies focusing on adolescent populations remain notably limited, with scarce data from Central Asia. Approximately half of the existing research has utilized PCR or DNA hybridization techniques, which show limitations; in contrast, our study not only implemented 16S rRNA sequencing to identify both known and novel bacteria but also used PICRUSt2 to investigate the predicted functional profiles of the microbial communities.

First, our study revealed intriguing patterns in oral microbial communities between caries and no caries groups. While alpha diversity showed no significant differences between the groups, beta diversity analysis demonstrated clear distinctions. This pattern suggests that caries may be more strongly associated with shifts in community composition rather than changes in overall species richness and evenness. According to a recent systematic review, many studies on the oral microbiome in adolescents reported no significant microbial diversity differences between caries positive and negative groups,^1^ while a meta-analysis by Butcher, et al.^22^ (2022) reported significant beta diversity differences in the oral microbiome of caries versus caries-free individuals, similar to our study. However, our observation of maintained alpha diversity differs from the findings of this report and other studies,^6,9,22,23^ which found reduced alpha diversity in caries-positive samples, suggesting that potential population-specific, age-specific, or methodological variations require further investigation. This finding supports the dental plaque hypothesis, implying that caries occurs due to changes in the relative proportions of resident bacteria rather than the presence or absence of specific pathogens.^2,23^ However, we admit that the absence of a significant difference in α diversity between the caries and no-caries groups might partly be due to variations in the sample sizes of the respective groups, as well as the relatively high mean DMFT (Decayed, Missing, Filled Teeth) value of 4.1.

Analyzing the oral microbiome composition, we identified several key bacterial taxa from the *Neisseria* and *Prevotella* genera. The Gammaproteobacteria class dominated the community structure, showing significant associations with dental caries, higher functional activity, and strong predictive value. Our findings differed from previous results, in which *Prevotella denticola*, *Scardovia wiggsiae*, *Streptococcus sobrinus*, and *Streptococcus mutans* were most frequently reported with higher abundance in adolescents with caries.^1^

Our findings revealed an association of the genus *Neisseria*, specifically *Neisseria elongata*, with adolescent caries. Indeed, the relationship between the *Neisseria genus* and dental caries remains controversial among various studies. Different research teams have reached opposing conclusions: some link *Neisseria* to caries,^24-26^ while others observe its greater presence in no-caries groups.^27,28^ It has been reported that specific *Neisseria* species can metabolize sugars to produce lactate, with some species capable of further converting lactate into stronger acids, indicating a potentially multifaceted role in oral health maintenance and disease development. Yet, our study revealed unexpected correlations between this taxon and non-carbohydrate metabolic pathways, suggesting a more complex role in oral microbiome function than previously recognized.

The *Prevotella* genus plays a significant role in dental caries development through its participation in complex microbial communities.^29^ Our results regarding this taxon are consistent with previous research.^28^ Various *Prevotella* species, including *Prevotella oris*, can utilize both sugar and protein metabolism, enabling their survival in both health and disease-associated conditions.^29,30^ In dentinal caries, *Prevotella oris* has been identified among the substantial core microbiota, demonstrating its adaptation to cariogenic environments.^30^ The species shows enhanced biofilm-forming abilities compared to other *Prevotella* species.^29^ Notably, *Prevotella oris* belongs to the core microbiome of the oral cavity of children, being present in at least 80% of children regardless of their parents’ periodontal status.^28-31^ While not a primary cariogenic pathogen, *Prevotella oris* contributes to caries progression through its metabolic versatility and ability to thrive in acidic conditions, participating in both structural maintenance and metabolic activities within dental biofilms.^32,33^

The role and interactions of Gammaproteobacteria class members in caries development remain poorly understood. Limited information exists about their contribution to caries formation, although Xu et al.^25^ (2014) revealed the significant presence of Pseudomonas, Acinetobacter, Enterobacteriaceae, and Cardiobacteriaceae, within bacterial networks associated with caries progression. Previous studies also showed that species from the Gammaproteobacteria class, such as *Haemophilus* and *Aggregatibacter* species of the *Pasteurellaceae* family, were linked to dental caries in children.^8^

Our findings indicate that shifts in the bacterial community are accompanied by specific changes in metabolic potential, particularly in pathways related to oxidative stress response and bacterial adaptation. Previous studies provide scarce comprehensive information on the metabolic function of caries-associated bacteria, commonly focusing on pathways involved in acid production from carbohydrates.^1^ Yet, our study revealed several strong correlations between key taxa and non-carbohydrate metabolic pathways, specifically glutathione metabolism, RNA degradation, and unsaturated fatty acid metabolism. The observed pathways suggest complex metabolic adaptations in the oral microbiome during caries progression, supporting specific bacterial survival and proliferation in acidic conditions.

Glutathione is an important antioxidant that helps maintain redox homeostasis. Existing research has suggested that an environment rich in glutathione, both reduced and oxidized forms, can support the survival of certain bacteria,^34^ including cariogenic bacteria. Glutathione imbalance, in turn, may lead to oxidative stress and inflammation in dental caries.^35^ For instance, the study by Dong-Hun Han et al.^35^ (2013) demonstrated significant associations between salivary glutathione levels and caries activity, with particular emphasis on reduced glutathione (GSH) as a key indicator of cariogenic conditions. Their findings showed that glutathione levels increased significantly with caries presence and bacterial load, specifically acidogenic bacteria such as *lactobacillus*. Our results expand on this by revealing significant correlations between glutathione metabolism pathways (p=4.1e-04) and caries-associated taxa. Specifically, this pathway correlated with prognostically significant bacteria including the genera *Prevotella* and *Neisseria*, and the class *Gammaproteobacteria*; the strong predictive ability of *Lactobacillus* observed in Figure 4 is also consistent with these findings. The dual role of glutathione as both an antioxidant response to bacterial activity and a potential biomarker for caries development is supported by these correlations, demonstrating its importance in complex metabolic interactions. This suggests that higher salivary glutathione levels may be associated with an increased presence of acidogenic and acid-tolerant bacteria, contributing to the progression of dental caries.

Studies have demonstrated that unsaturated fatty acids play a pivotal role in dental caries through multiple mechanisms.^36^ The major unsaturated fatty acids, mainly oleic and linolenic acid, show remarkable antibiofilm activity against *Streptococcus mutans* at low concentrations by interfering with several virulence factors.^37^ These fatty acids work by blocking bacterial primary adhesion, disrupting cell-to-cell communication, inhibiting extracellular polysaccharide production, and reducing acid production.^32,38^ They can act as micelles surrounding oral bacteria, preventing adhesion to tooth enamel. Notably, while effectively targeting these virulence factors, the fatty acids maintain bacterial viability and show no cytotoxicity to human oral cells, suggesting their potential as safe and natural alternatives for caries prevention.^33,38,39^ On the other hand, unsaturated fatty acids, which are crucial molecular components with diverse functions across biological systems and play critical roles in cellular structure, energy storage, signaling, and environmental adaptation, can also be used by favorable bacteria to thrive. Notably, microorganisms can modify fatty acid composition to survive extreme conditions, adapting to temperature, pH, and environmental stress.^37,38^ Thus, according to our findings, activation of this pathway appear to be compensatory and protective against the growth of cariogenic bacteria.

The RNA degradation pathway represents a complex molecular mechanism in which the RNA degradosome functions as a critical enzymatic system. This multiprotein complex initiates RNA recognition and performs precise molecular clearance, strategically breaking down RNA molecules using RNase E, PNPase, RhlB, and enolase.^38^ This pathway allows bacteria to rapidly modify genetic expression, manage cellular resources, and respond dynamically to environmental challenges. This mechanism provides a crucial survival strategy for cariogenic bacteria, facilitating quick genetic reprogramming, metabolic flexibility, and resilience in challenging microenvironments in the oral cavity. The RNA degradosome operates not as a destructive system but as an adaptive mechanism, supporting bacterial survival and evolutionary success across diverse ecological niches.^38,39^

These findings reveal the functional capacity of microbial communities beyond simple taxonomic identification, providing information on how bacterial populations may adapt, compete, and coexist in the oral environment. Understanding these relationships is essential for comprehending oral microbiome dynamics and their broader health implications, as they reveal how specific bacterial groups utilize distinct metabolic activities during caries progression.

This study shows several limitations that should be considered when interpreting the results. One of the limitations is that we did not differentiate between the oral microbiome associated with active versus treated caries. Our study was designed to assess overall microbial patterns in relation to caries status. Future studies focusing specifically on the decayed component (D) of the DMFT index will be better suited to address this question. Another limitation is that the sample size, precisely the limited control group, may have impacted our ability to fully account for confounding factors. Additionally, our cross-sectional findings provide a foundation for understanding caries-associated microbial signatures in this population. Considering the unique characteristics of the oral microbiome in adolescents, it may be important to monitor changes over time. Investigation of longitudinal shifts in the oral microbiome of adolescent subjects could provide valuable insights into the development and progression of dental caries. The 16S rRNA sequencing, while informative for taxonomic profiling, does not provide the same depth of functional information as metagenomic approaches. Additionally, the metabolic pathway analysis relies on predictive algorithms based on taxonomic data rather than direct functional gene measurements. These methodological constraints should be considered, and future studies with larger cohorts and more comprehensive sequencing techniques should be performed to confirm the patterns observed in this investigation.

## Conclusions

In conclusion, our study highlights specific oral microbiome patterns associated with dental caries in Kazakhstani adolescents, revealing interactions between key bacterial taxa and their metabolic pathways. We found that bacterial species such as *Neisseria*, *Prevotella*, and *Gammaproteobacteria* were linked with processes such as glutathione metabolism, RNA degradation, and unsaturated fatty acid metabolism, which may support their persistence and contribute to microbial imbalance associated with caries risk. These findings provide new insights into the oral microbiome of Kazakhstani adolescents and underscore the importance of microbial balance in sustaining oral health.
